# Surgical Cases

**Published:** 1866-03

**Authors:** Geo. K. Amerman

**Affiliations:** Attending Surgeon to the Chicago City Hospital


### ARTICLE X.

### SURGICAL CASES.

BY GEO. K. AMERMAN, Attending Surgeon to the Chicago City Hospital.

CASE I. *Strangulated Femoral Hernia—Operation—Recovery.*

Miss G., aged 25, was attacked on Thursday, March 16th, with pain in the lower part of the abdomen, nausea, and vomiting. In the evening she took a dose of senna, prescribed by a friend, after which the pain and vomiting increased, so that, on Friday evening, Dr. HURLBUT, the family physician, was called. He at once recognized the true state of the case, and made a careful but ineffectual effort to reduce the tumor. The pain and vomiting continuing, I was invited by Dr. H. to see the patient, with a view of operating if the taxis failed after another patient trial. At this time, the case presented all the usual phenomena of a strangulated femoral hernia. The tumor was not large, but firm and closely constricted. Dr. H. administered chloroform to complete anæsthesia, and we made an unsuccessful effort at reduction. A large dose of opium was ordered for the night, and the operation determined upon early the next morning.

Saturday, March 18.—No change in the character of the tumor or symptoms since yesterday. Vomiting and pain continue; bowels constipated from the first; pulse 96. Decided to operate, and, with the assistance of Dr. Bogue, proceeded in the usual way to expose the sac. It was found unusually distended with dark-colored serum, and adhering to its walls everywhere, a loop of intestine perfectly black but glistening and firm. The stricture was divided, and the intestine left lying in the sac. A couple of sutures in the upper part of the wound, the remainder left open, with simple cold water dressings, completed the operation.

The opium treatment was adopted, and, after a very protracted convalescence, contrary to our expectations, the patient fully recovered, still being the subject of a small-sized reducible femoral hernia necessitating the constant use of a truss.

Case II. *Strangulated Femoral Hernia—Operation—Death on the fourth day.*

Mrs. G., aged 66, for 26 years the subject of a large, irreducible femoral hernia, was taken on Thursday, May 18th, with vomiting, diarrhœa, and pain in the bowels. As such attacks were of common occurrence, her friends resorted to simple domestic means, and on Friday morning the vomiting and diarrhœa ceased, but the pain and tenderness continued.

On Friday evening, Dr. Hurlbut, the family physician, was called. He at once recognized the difficulty, and made an unsuccessful effort at reduction. On Saturday evening, I was invited by Dr. H. to see the case. She then had all the usual symptoms of strangulated hernia. The tumor was large, hard, painful, and dull on percussion. Pulse 100 and feeble.

It was decided to administer an anæsthetic, try taxis, and, if unsuccessful, operate. Dr. H. administered chloroform to complete anæsthesia, and, after a patient trial to reduce, without the slightest effect, an incision was made through the external parts, the sac opened, and a large mass of omentum exposed. It presented a perfectly natural appearance, except, perhaps, rather more injection and less glistening. The stricture was divided and about two-thirds of the mass returned, the remainder left lying in the sac. A few sutures and simple dressings to the wound completed the operation.

The opium treatment was adopted, and everything progressed favorably until the morning of the fourth day, when she began to be delirious and show signs of prostration. Brandy, beef-tea, and quinine were given during the day, but she sank rapidly and died in the evening.

*Remarks.*—There are one or two interesting features connected with the above cases, which may be noted and made useful in future cases of a similar character. In Case I., strangulation occurred on Thursday, March 16th, and on Saturday, March 18th, at the time of the operation, the intestine was apparently gangrenous, though not softened; whilst in Case II., at the time of the operation, (the same time having elapsed after the occurrence of the strangulation as in Case I.,) the omentum was found almost perfectly natural. The symptoms before the operation were as urgent in the one as in the other, and, indeed, if external circumstances were any guide, we should have inferred a much more favorable condition of the parts in the first case than in the second. The age of the patient, the length of time the hernia had existed, and the size of the tumor were all favorable in Case I., and, hence, we believe, that in all cases of strangulated hernia, the operation should be performed early, without any regard to the apparent urgency of the symptoms. As soon as one careful, fair, and patient effort to reduce fails, where all the surroundings are favorable, the chances are that nothing short of an operation will save your patient, and the sooner that is resorted to the better are the chances of a successful result.

Secondly. The apparent condition of the hernial contents cannot always be relied on in forming a prognosis. In Case I. the intestine was found quite black and, to all appearances, almost certain to slough, and yet the patient made a good recovery; whilst in Case II., although the omentum presented a perfectly natural appearance, death followed on the fourth day. The age of the patient, the length of time the hernia has existed, and the size of the tumor are all to be taken into the account, and, along with the condition of the contents of the sac, will enable us to form a reasonable prognosis in nearly every case.

